# Comparable outcomes of outpatient remdesivir and sotrovimab among high-risk patients with mild to moderate COVID-19 during the omicron BA.1 surge

**DOI:** 10.1038/s41598-024-56195-y

**Published:** 2024-03-05

**Authors:** Supavit Chesdachai, Christina G. Rivera, Kristin C. Cole, Hilary R. Teaford, Maria L. Gonzalez Suarez, Jennifer J. Larsen, Ravindra Ganesh, Sidna Tulledge-Scheitel, Raymund R. Razonable

**Affiliations:** 1 Division of Public Health, Infectious Diseases, and Occupational Medicine, Mayo Clinic, 200 First St SW, Rochester, MN 55905 USA; 2 Department of Pharmacy, Mayo Clinic, Rochester, MN USA; 3 Department of Quantitative Health Sciences, Mayo Clinic, Rochester, MN USA; 4 Division of Nephrology and Hypertension, Mayo Clinic, Rochester, MN USA; 5 Department of Nursing, Mayo Clinic, Rochester, MN USA; 6 Department of General Internal Medicine, Mayo Clinic, Rochester, MN USA; 7 Department of Community Internal Medicine, Mayo Clinic, Rochester, MN USA

**Keywords:** COVID-19, Outcome, Remdesivir, SARS-2-CoV, Sotrovimab, Outcomes research, Viral infection

## Abstract

Studies conducted prior to SARS-CoV-2 Omicron demonstrated that sotrovimab and remdesivir reduced hospitalization among high-risk outpatients with mild to moderate COVID-19. However, their effectiveness has not been directly compared. This study examined all high-risk outpatients with mild to moderate COVID-19 who received either remdesivir or sotrovimab at Mayo Clinic during the Omicron BA.1 surge from January to March 2022. COVID-19-related hospitalization or death within 28 days were compared between the two treatment groups. Among 3257 patients, 2158 received sotrovimab and 1099 received remdesivir. Patients treated with sotrovimab were younger and had lower comorbidity but were more likely to be immunocompromised than remdesivir-treated patients. The majority (89%) had received at least one dose of COVID-19 vaccine. COVID-19-related hospitalization (1.5% and 1.0% in remdesivir and sotrovimab, respectively, p = .15) and mortality within 28 days (0.4% in both groups, p = .82) were similarly low. A propensity score weighted analysis demonstrated no significant difference in the outcomes between the two groups. We demonstrated favorable outcomes that were not significantly different between patients treated with remdesivir or sotrovimab.

## Introduction

The coronavirus disease 2019 (COVID-19) caused by the severe acute respiratory syndrome coronavirus 2 (SARS-CoV-2) has led to significant morbidity and mortality worldwide^1^. Numerous therapeutic trials have focused on the prevention of severe outcomes, particularly in high-risk individuals with mild to moderate diseases, with the goal of alleviating complications of severe illnesses, hospitalization, and death^2^.

Monoclonal antibodies (mAbs) targeting the spike protein of SARS-CoV-2 were the first therapeutic agents that received emergency use authorizations (EUA) for use in managing mild to moderate COVID-19. Unfortunately, the effectiveness of each mAb was short-lived due to the rapid emergence of SARS-CoV-2 variants with spike mutations that limited the neutralization properties of the mAbs^3^.

Sotrovimab was considered as one of the most effective mAbs which retains in vitro efficacy against many variants^4^. Multicenter, double-blind, placebo-controlled trial demonstrated that sotrovimab substantially reduced the risk of disease progression among high-risk patients exhibiting mild-to-moderate symptoms^5^. This clinical trial led to the EUA for sotrovimab from the United States Food and Drug Administration (FDA) in May 2021^6^. Sotrovimab was the only mAb that was authorized for clinical use in high-risk individuals with mild-to-moderate COVID-19 in the outpatient setting or those who were admitted for non-COVID-19 reasons and were subsequently diagnosed with mild-to-moderate COVID-19 when the Omicron B.1.1.529 variant was the predominant strain, since casirivimab-imdevimab and bamlanivimab-etesevimab were no longer effective. Due to limited supply of mAb treatment, many centers including ours preferentially allocated sotrovimab to the most vulnerable patients such as immunocompromised hosts.

In December 2021, intravenous remdesivir gained approval for use as an outpatient therapeutic agent. The randomized, double-blind, placebo-controlled trial demonstrated that an outpatient 3-day course of remdesivir resulted in diminished rates of hospitalization or death by 87% compared to placebo^7^. As a result, during the surge of the Omicron variant (B.1.1.529) from January to March 2022, sotrovimab and remdesivir were the two primary outpatient therapeutic agents for high-risk patients with mild to moderate COVID-19. The supplies of oral antivirals such as ritonavir-boosted nirmatrelvir and molnupiravir were limited at that time.

There is very limited data comparing the efficacy of sotrovimab and remdesivir for preventing hospitalization and death^8^. Accordingly, we aim to compare the real-world efficacy of remdesivir and sotrovimab for the treatment of mild to moderate COVID-19. While the results of this study do not reflect the current COVID-19 variant and its therapeutic landscape, the study provides evidence of the comparable efficacy of a small-molecule antiviral drug and a large-molecule antibody product for treatment of high-risk patients with mild to moderate COVID-19.

## Methods

### Study design and population

This is a retrospective study that included all high-risk, adult (age 18 years or older) patients who developed mild to moderate COVID-19 and received either a single dose of sotrovimab (500 mg dose as a single infusion) or a 3-day course of remdesivir (200 mg infusion on day 1 followed by 100 mg on day 2 and 3) as an outpatient therapy during the SARS-CoV-2 Omicron variant (B.1.1.529) surge from January 1 to March 15, 2022 at Mayo Clinic Rochester, Florida, Arizona, and Mayo Clinic Health System in southern Minnesota, northeastern Iowa, and western Wisconsin. The eligible patients were identified from the Mayo Clinic electronic health records. Exclusion criteria were (1) Patients who were hospitalized on the same day of sotrovimab or remdesivir infusion; (2) Patients who declined authorization to use their medical record for research purpose based on their state statute.

The Mayo Clinic Institutional Review Board has reviewed and approved the study (Study IRB number 20-012975). The research has been performed in accordance with the Declaration of Helsinki. The study was granted an exemption from patient consent by the Mayo Clinic Institutional Review Board as the study collected and analyzed secondary clinical data without patients contact or any intervention and it did not include factors necessitating patient consent.

### Infusion therapy program

In November 2020, Mayo Clinic launched the Monoclonal Antibody Treatment Program (MATRx) offering mAbs therapies to high-risk individuals with mild to moderate COVID-19 in an outpatient setting^9^. Additionally, this structure was adapted to facilitate outpatient intravenous remdesivir therapy. All patients who tested positive for COVID-19 were screened using Monoclonal Antibody Screening Score (MASS), which is composed of the FDA EUA eligibility criteria for mAbs therapy issued in November 2020^10^ (Component of MASS can also be found in Table 1). Patients who had MASS score ≥ 2 were defined as high-risk patients and eligible for outpatient therapies.

**Table 1:** Baseline demographic and clinical characteristics of high-risk patients who received sotrovimab or remdesivir for mild to moderate coronavirus disease 2019.

	Total (N = 3257)	Remdesivir (N = 1099)	Sotrovimab (N = 2158)	p value
Age (years), Median (IQR)	64 (49, 73)	69 (60, 76)	60 (41, 71)	< 0.001
Female	1862 (57.2%)	567 (51.6%)	1295 (60.0%)	< 0.001
Race				0.006
White	3029 (93.0%)	1037 (94.4%)	1992 (92.3%)	
American Indian/Alaskan Native	18 (0.6%)	8 (0.7%)	10 (0.5%)	
Asian	47 (1.4%)	12 (1.1%)	35 (1.6%)	
Black or African American	99 (3.0%)	20 (1.8%)	79 (3.7%)	
More than one race	18 (0.6%)	6 (0.5%)	12 (0.6%)	
Native Hawaii/Pacific Islander	4 (0.1%)	0 (0.0%)	4 (0.2%)	
Other	21 (0.6%)	4 (0.4%)	17 (0.8%)	
Unknown	21 (0.6%)	12 (1.1%)	9 (0.4%)	
Ethnicity				0.73
Hispanic or Latino	135 (4.1%)	44 (4.0%)	91 (4.2%)	
Not Hispanic or Latino	3084 (94.7%)	1040 (94.6%)	2044 (94.7%)	
Unknown	38 (1.2%)	15 (1.4%)	23 (1.1%)	
Mean BMI (SD)^a^	31.3 (7.3)	32.3 (7.8)	30.7 (7.0)	< 0.001
Median serum creatinine (IQR)^b^	1.0 (0.8, 1.2)	1.0 (0.8, 1.2)	1.0 (0.8, 1.2)	0.27
Median eGFR (IQR)^b^	77.0 (60.3, 93.4)	75.1 (59.2, 91.1)	78.5 (60.8, 95.1)	< 0.001
Received tixagevimab/cilgavimab	7 (0.2%)	1 (0.1%)	6 (0.3%)	0.28
Number of COVID vaccines prior to start, Median (IQR)	3 (2, 3)	3 (2, 3)	3 (2, 3)	0.012
0	368 (11.3%)	109 (9.9%)	259 (12.0%)	
1	94 (2.9%)	18 (1.6%)	76 (3.5%)	
2 +	2795 (85.8%)	972 (88.4%)	1823 (84.5%)	
Days from last vaccination to the initiation of drug therapy, Median (IQR)	129 (95, 163)	116 (90, 150)	137 (98, 168)	< 0.001
Charlson Comorbidity Index, Median (IQR)	3 (1, 6)	4 (2, 7)	3 (1, 6)	< 0.001
MASS Score, Median (IQR)	5 (4, 7)	6 (4, 8)	5 (3, 7)	< 0.001
MASS components				
Age ≥ 65	1626 (49.9%)	740 (67.3%)	886 (41.1%)	< 0.001
BMI ≥ 35	1090 (33.5%)	436 (39.7%)	654 (30.3%)	< 0.001
Cardiovascular disease	1057 (32.5%)	519 (47.2%)	538 (24.9%)	< 0.001
Chronic respiratory disease	635 (19.5%)	335 (30.5%)	300 (13.9%)	< 0.001
Chronic kidney disease stage IV/V	183 (5.6%)	64 (5.8%)	119 (5.5%)	0.72
Diabetes mellitus	888 (27.3%)	425 (38.7%)	463 (21.5%)	< 0.001
Hypertension	1577 (48.4%)	732 (66.6%)	845 (39.2%)	< 0.001
Immunocompromised status	1353 (41.6%)	332 (30.2%)	1021 (47.3%)	< 0.001
Pregnancy	306 (9.4%)	0 (0.0%)	306 (14.2%)	< 0.001

This table demonstrated baseline demographic of this population. Patients treated with sotrovimab were younger and had lower comorbidity but were more likely to be immunocompromised than remdesivir-treated patients.

^a^Available in 2704 (933 remdesivir and 1771 sotrovimab).

^b^Available in 2686 (974 remdesivir and 1712 sotrovimab).

High-risk patients were offered either a single dose of sotrovimab or 3-day course of remdesivir. The period between January 1 and March 15, 2022, marked the timeframe during which both sotrovimab and remdesivir treatments were concurrently available and administered. Sotrovimab was then replaced by bebtelovimab after March 15, 2022, due to the emergence of Omicron variants that were not effectively neutralized by sotrovimab.

### Definitions

COVID-19 was confirmed by a positive SARS-CoV-2 polymerase chain reaction or antigen test, within 5 (for remdesivir) or 7 days (for sotrovimab) of symptom onset. The interval between the onset of symptoms and the administration of the drug was strictly governed by the criteria outlined in the EUA. Home antigen tests were deemed acceptable; patients with positive results were instructed to send the photo of the positive home antigen test to their primary care team, who would then document the test outcome in the electronic medical record. Mild to moderate COVID-19 was defined as mild COVID-19 symptoms with or without clinical or radiographic evidence of lower respiratory tract diseases (oxygen saturation ≥ 94%) and absence of features for severe or critical illness^11,12^.

### Outcome measure

The primary outcome of the study was composite rate of COVID-related hospitalization or death within 28 days after treatment initiation. This primary outcome was assessed for the whole population and compared between patients who received sotrovimab and those who received remdesivir. In addition, risk factors for hospitalization or death within 28 days for this population were investigated.

### Statistical analysis

Means and standard deviations (SD) or medians and interquartile ranges (IQRs) were used to summarize continuous variables. Frequencies (N) and percentages (%) were used for categorical data. Clinical characteristics were compared between groups using Wilcoxon rank sum tests for continuous data and either Chi‐square or Fisher's exact tests for categorical data. Univariable logistic regression was used to assess associations between the treatment group and 28-day hospitalization or death. As a sensitivity analysis, we also used propensity score (PS) weighting to adjust for imbalances in the measured baseline characteristics between those receiving remdesivir and sotrovimab. PS values were estimated using a multivariable logistic regression model, where remdesivir was the outcome, and variables in Table 1 were the covariates. The PS weights were defined as 1/PS for remdesivir patients and 1/(1-PS) for sotrovimab patients. The weights in each group were then divided by the respective mean weight for that group, so the sum of the weights was equal to the original group sample size. Imbalances in baseline characteristics was assessed using standardized differences, where a standardized difference < 0.10 represents negligible imbalance. Weighted logistic regression was then used to assess the association between treatment and 28-day hospitalization or death. All analyses were performed using SAS version 9.4 software (SAS Institute, Inc; Cary, NC).

## Results

### Baseline demographics

A total of 3257 high-risk patients with mild to moderate COVID-19 received outpatient COVID-19 directed therapies during the study period: 2158 (66.3%) received sotrovimab and 1099 (33.7%) received remdesivir. The median age was 64 [IQR 49, 73]; 1862 (57.2%) were female and 3029 (93.0%) were white. Patients treated with sotrovimab were younger (median age of 60 compared to 69 years in remdesivir group). The mean BMI was 31.3 (SD 7.3).

Most patients (N = 2889, 88.7%) had received at least one dose of a COVID-19 vaccine, and the median number of vaccines received was 3 [IQR 2, 3]. The median duration from the last vaccination to the initiation of drug therapy was 129 days [IQR 95, 163]. The median MASS and Charlson Comorbidity Index (CCI) were 5 [IQR 4, 7] and 3 [IQR 1, 6], respectively.

Forty two percent of the cohort (N = 1353) were considered as having immunocompromised status. Patients in sotrovimab group were more likely to be immunocompromised than those who received remdesivir (47.3% versus 30.2%). A detailed baseline demographic can be found in Table 1.

### Clinical outcomes

**Figure 1:**
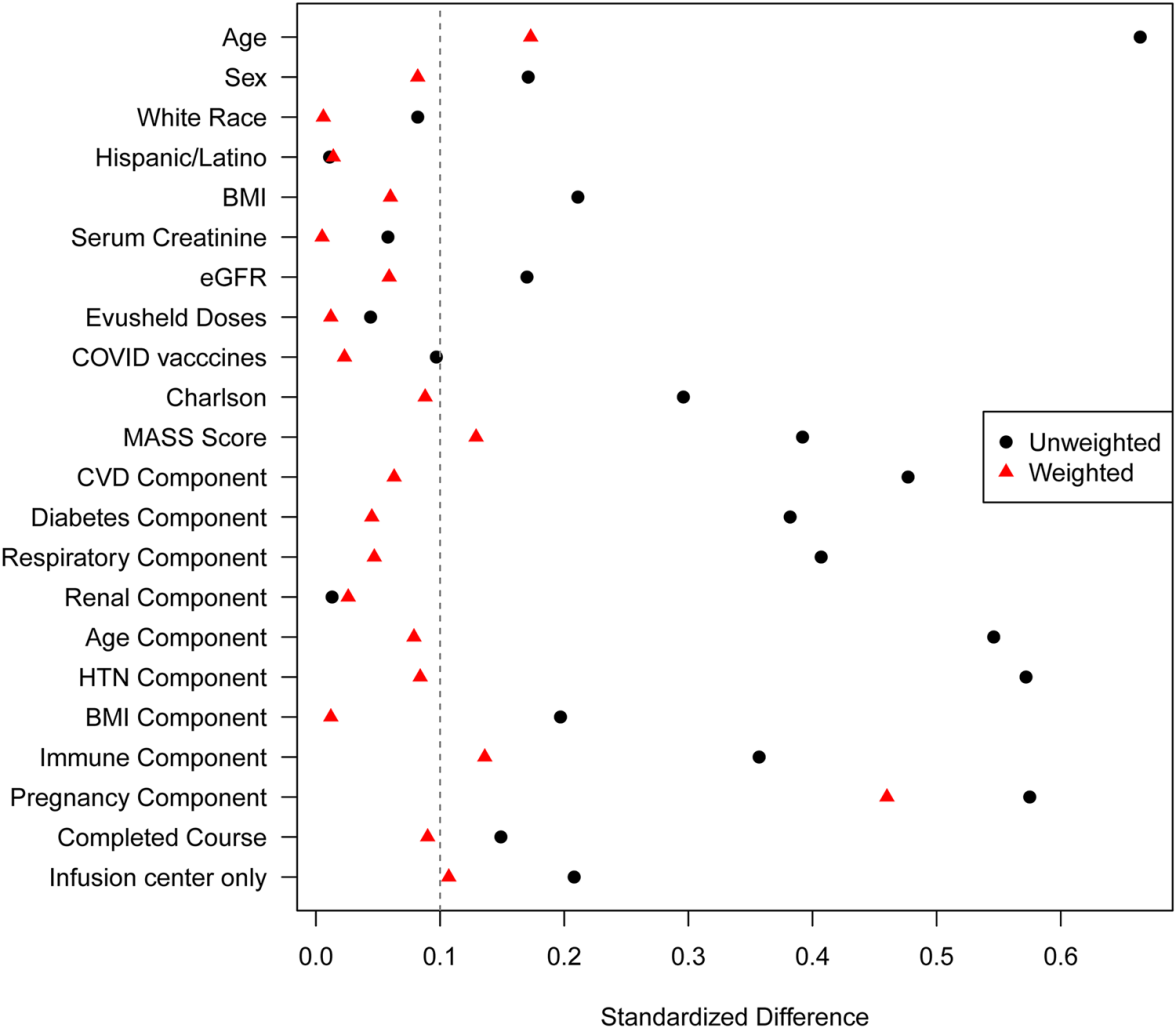
Standardized differences for each baseline characteristic used in the derivation of the propensity scores in the unweighted and weighted cohorts. Imbalances in baseline characteristics was assessed using standardized differences, where a standardized difference < 0.10 represents negligible imbalance.

The overall rate of COVID-19-related hospitalizations or death within 28 days was 1.5% (n = 49). COVID-19 related hospitalizations were comparable between the two groups, 21 (1.0%) in the sotrovimab and 17 (1.5%) in the remdesivir groups (p = 0.15). The hospitalization rates observed in each group did not show a statistically significant difference across the months during the study period. Likewise, the 28-day all-cause mortality were similar between the two treatment groups, at 0.4% in both groups (p = 0.82). After reducing the imbalance in baseline patient characteristics by PS weighting (Fig. 1), we demonstrated no significant difference in the rates of COVID-19-related hospitalization or death within 28 days between these two groups (sotrovimab 1.5% vs 1.6% remdesivir, p = 0.79).

### Risk factors for hospitalization and mortality

**Figure 2:**
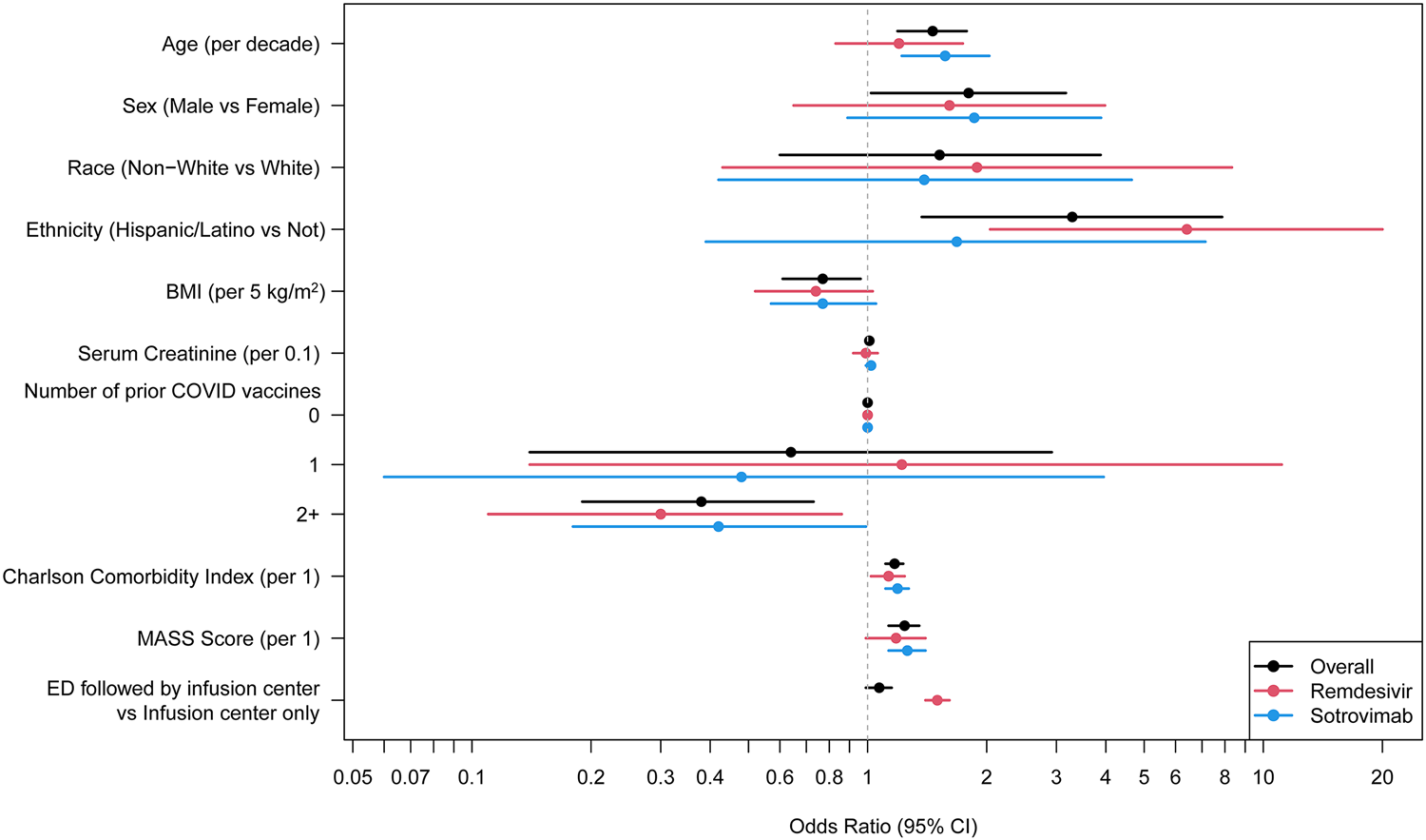
Univariate risk factor analysis for hospitalization or death overall and in each treatment group. Older age, Hispanic or Latino ethnicity, obesity, unvaccinated status, and higher co-morbidity were associated with hospitalization or mortality within 28 days.

Univariable analysis revealed several factors that were associated with hospitalization or mortality within 28 days, including older age, Hispanic or Latino ethnicity, obesity, unvaccinated status, and higher co-morbidity as measured by high MASS score and a higher CCI. However, due to the limited number of outcomes, conducting a multivariable analysis was not feasible. Figure 2 illustrates the results of the univariable risk factor analysis for hospitalization or death within each treatment group.

## Discussion

Our study indicated that there were no significant differences in the rates of COVID-19-related hospitalization or death within 28 days between sotrovimab and remdesivir despite variations in baseline patient characteristics. The composite rate of COVID-19-related hospitalization or death overall and for both the treatment groups was relatively low, reflecting the clinical utility of these therapeutic agents in mitigating severe outcomes in high-risk individuals with mild to moderate COVID-19.

Our study's findings align with the results from a previous investigation; Piccicacco et al., showed that both remdesivir and sotrovimab had comparable effects in preventing hospitalization and emergency department visits among high-risk individuals^8^. Notably, in both studies, sotrovimab was preferentially given to immunocompromised patients, and they are theoretically less able to mount an immune response to the natural SARS-CoV-2 infection.

It is also essential to consider the factors influencing hospitalization in our study, even in a cohort with a high vaccination rate and access to effective outpatient therapies. Our univariate analysis identified some risk factors that were associated with hospitalization and mortality including an older age, being Hispanic or Latino, obesity, unvaccinated status, and high degree of medical comorbidity. It is worth noting that pregnant patients preferred treatment with sotrovimab, as there was limited data on the safety of remdesivir for this population at the time of this study. These observations are not surprising as they have previously been demonstrated by prior studies^13^. Importantly, these identified risk factors represent a subset of individuals for whom the threat of hospitalization and mortality persists, despite the availability of targeted therapies. Recognizing and addressing the unique vulnerabilities of this group is essential in the ongoing effort to enhance COVID-19 management strategies and minimize adverse outcomes.

Fundamental differences in the mechanism of action between remdesivir and sotrovimab are worth noting. Remdesivir operates as a small molecule antiviral drug, inhibiting viral RNA synthesis, as it serves as a nucleotide analog prodrug that halts viral replication if it gets incorporated during viral nucleic acid synthesis^14^. On the other hand, sotrovimab is a passively administered monoclonal antibody targeting the entry of the SARS-CoV-2 at the spike protein structure^15^. In addition to the differences in mechanism of action, there are logistical differences in drug administration. Notably, the ease of administration of a single dose monoclonal antibody, like sotrovimab, is an advantage when compared to the multiple daily doses required for remdesivir. However, the rapidly changing viral variants with mutations in spike protein that resulted in reduced binding affinity of monoclonal antibody is a significant challenge to the therapeutic lifespan of the COVID-19 anti-spike monoclonal antibodies^16^.

While sotrovimab and other mAbs are no longer available for clinical use, our findings provided real-world data on comparability of outcomes between different classes of therapeutic agents and variations in treatment strategies. Furthermore, this study underscores the pressing need for effective strategies to prevent severe diseases among the most vulnerable populations. Our study has several limitations. First, the decision to administer remdesivir or sotrovimab may have been influenced by shared decision-making between providers and patients, which was not entirely accounted for in the analysis. Factors that could influence decision-making also included the drug supply availability, distance to the infusion center, provider and patient preferences, number of infusions, pregnancy status and baseline renal or hepatic function. Second, the limited number of events in our study constrained our ability to conduct a more extensive multivariable analysis to assess the independent effects of risk factors on outcomes. Third, despite employing propensity score weighting, we could not achieve perfect balance between the two groups in terms of clinical characteristics, specifically regarding vaccination rates and the presence of comorbidities. Fourth, the absence of a control group (untreated) hinders the assessment of each medication's effectiveness as compared to no treatment. Finally, the retrospective nature of this single-center study introduces the potential for confounding variables that may be difficult to ascertain.

In conclusion, our study demonstrated the comparable real-world efficacy of remdesivir and sotrovimab for the treatment of high-risk patients with mild to moderate COVID-19, despite differences in baseline comorbidities and other characteristics. Both the antiviral and antibody-based therapies were similarly effective in reducing the risk of hospitalization and mortality of patients with mild to moderate COVID-19 during SARS-CoV-2 Omicron variant (B.1.1.529) surge.

## Data Availability

The datasets generated during and/or analyzed during the current study are not publicly available due to HIPPA protection but are available from the corresponding author on reasonable request.

## Abbreviations

CCI: Charlson Comorbidity Index
COVID-19: Coronavirus disease 2019
BMI: Body mass index
eGFR: Estimated glomerular filtration rate
EUA: Emergency use authorizations
FDA: Food and Drug Administration
IQRs: Interquartile ranges
mAbs: Monoclonal antibodies
MASS: Monoclonal antibody screening score
MATRx: Monoclonal antibody treatment program
N: Frequencies
PS: Propensity score
SARS-CoV-2: Severe acute respiratory syndrome coronavirus 2
SD: Standard deviations
